# Association Between Steroid-Sparing Therapy and the Risk of Perianal Fistulizing Complications Among Young Patients With Crohn Disease

**DOI:** 10.1001/jamanetworkopen.2020.7378

**Published:** 2020-06-09

**Authors:** Jeremy Adler, Chun Chieh Lin, Samir K. Gadepalli, Kevin J. Dombkowski

**Affiliations:** 1 Division of Pediatric Gastroenterology, Department of Pediatrics, University of Michigan, Ann Arbor; 2 Susan B. Meister Child Health Evaluation and Research Center, University of Michigan, Ann Arbor; 3 Institute for Healthcare Policy and Innovation, University of Michigan, Ann Arbor; 4 Department of Neurology, University of Michigan, Ann Arbor; 5 Division of Pediatric Surgery, Department of Surgery, University of Michigan, Ann Arbor

## Abstract

**Importance:**

Perianal fistulizing complications (PFCs) develop in 15% to 30% of patients with Crohn disease (CD), are difficult to treat, worsen quality of life, increase cost of care, and commonly recur. Evidence-based strategies to prevent PFCs are lacking.

**Objectives:**

To investigate the effectiveness of medical therapy for reducing risk of PFCs among young people with CD and to test the hypothesis that steroid-sparing therapy (SST) use would be associated with reduced risk of PFC development.

**Design, Setting, and Participants:**

In this comparative effectiveness analysis of commercial administrative claims from January 1, 2001, through June 30, 2016, patients who did or did not initiate SST were matched via propensity score to adjust for all available confounders. Using Optum’s Clinformatics Data Mart, a deidentified database of US commercial administrative claims, all patients aged 5 to 24 years with CD (January 1, 2001, through June 30, 2016) were identified. The index date was the CD diagnosis date. Patients with PFCs or SST use at or before CD diagnosis were excluded. The dates of analysis were October 2017 to February 2020.

**Exposures:**

The primary exposure of interest was SST initiation, including immunomodulators and/or anti–tumor necrosis factor α (anti-TNFα) medications, initiated before either PFC development or the end of the study period.

**Main Outcomes and Measures:**

The primary outcome was PFC development. Propensity score matching was used to balance baseline characteristics. Cox proportional hazards multivariable regression analyses were used to estimate hazard ratios (HRs) with 95% CIs for PFC development.

**Results:**

Among 2214 young people with CD without PFCs identified, the mean (SD) age at CD diagnosis was 17.0 (4.5) years, and 1151 (52.0%) were male. Among the cohort, 1242 patients (56.1%) initiated SST before PFC development or the end of 24-month follow-up. After propensity score matching, 972 patients remained in each treatment group. Overall, 384 of 1944 (19.8%) developed PFCs within 2 years of the index date. The use of SST was associated with a 59% decreased risk of PFC development (hazard ratio [HR], 0.41; 95% CI, 0.33-0.52; *P* < .001) in 2 years compared with no SST use. Among those who developed PFCs, 55% fewer SST users underwent ostomy than SST nonusers. The use of immunomodulators alone, anti-TNFα alone, and combination therapy was associated with 52% (HR, 0.48; 95% CI, 0.37-0.62; *P* < .001), 47% (HR, 0.53; 95% CI, 0.36-0.78; *P* = .001), and 83% (HR, 0.17; 95% CI, 0.09-0.30; *P* < .001) reductions in the risk of 2-year PFC development, respectively, compared with no SST use.

**Conclusions and Relevance:**

In this study, PFC development was common among young patients with CD. The use of SST was lower than expected. Compared with no SST, patients who initiated SST were 59% less likely to develop PFCs and fewer underwent ostomy. These results indicate that PFCs may be preventable and emphasize the importance of considering SST for all patients with CD.

## Key Points

**Question:**

Does steroid-sparing therapy for Crohn disease reduce the risk of developing perianal fistulizing complications?

**Findings:**

In this comparative effectiveness analysis 2214 young people with Crohn disease without perianal fistulizing complications were matched via propensity score. Almost 20% developed perianal fistulizing complications within 2 years of Crohn disease diagnosis. Steroid-sparing therapy use was statistically significantly associated with a 59% reduction in perianal fistulizing complications, and fewer underwent ostomy among those who developed perianal fistulizing complications and who had been previously treated with steroid-sparing therapy.

**Meaning:**

Steroid-sparing therapy should be considered for treatment of Crohn disease to reduce the risk of perianal fistulizing complications.

## Teaser

This comparative effectiveness analysis of commercial administrative claims uses propensity score matching to investigate the effectiveness of medical therapy for reducing risk of perianal fistulizing complications among young people with Crohn disease.

## Introduction

Crohn disease (CD) is a chronic condition, with estimated prevalence in the Western world ranging from 250 to 1300 cases per 100 000 individuals.^1,2^ It can cause severe destructive transmural intestinal inflammation,^3,4^ which may create fistulas that penetrate through the bowel wall. Fistulas develop more commonly among those with childhood-onset CD (30%) vs adult-onset CD (15%-20%).^5,6,7,8^ Fistulas provide a path for feculent material to invade adjacent tissues, causing septic complications or soiling of the skin via cutaneous openings.^5,9,10^ The most common site for fistula development is in the perianal region, including perianal fistulas, perirectal abscesses, rectovaginal fistulas, and fistulas to the scrotum or labia, collectively referred to as perianal fistulizing complications (PFCs).^5,9,11^ These complications can be severe, causing major negative consequences, including reduced quality of life (QOL), long-term detrimental health consequences, and increased health care expenditures.^11,12,13,14,15^

Despite improvements in medical therapies, PFCs remain difficult to treat and commonly reoccur.^5,16,17,18^ About 70% of patients with PFCs eventually undergo surgical interventions, which often provide only temporary relief.^6,17,19^ Despite all available medical and surgical interventions, 8% to 19% of patients with PFCs eventually undergo permanent diverting ostomy.^17,19,20^ Prevention of PFC development through effective medical treatments could lead to better outcomes. However, the epidemiology and progression of PFCs are not well understood, and evidence for effective preventive strategies is lacking.

Steroid-sparing therapy (SST), including immunomodulators and anti–tumor necrosis factor α (anti-TNFα) medications, improves many CD outcomes, but it remains to be determined whether PFCs are preventable and, if so, which medical therapies are most efficacious.^21,22,23^ We hypothesized that SST use would be associated with prevention of PFCs among patients who were free from such complications at CD diagnosis. Prevention of PFCs could lead to improved QOL and reduced health care use for approximately one-fourth of patients with CD.^2,24,25,26^ With that in mind, we designed a study to assess the effectiveness of SST for preventing PFCs among children and young adults with newly diagnosed CD. We chose this population because of their high incidence of PFCs.^5,6,7,8^

## Methods

### Data Source

In this propensity score–matched retrospective analysis, data were analyzed from Optum’s Clinformatics Data Mart, a statistically deidentified database of US commercial administrative health claims that includes inpatient, outpatient, pharmacy, and laboratory administrative claims for individuals with commercial health insurance (January 1, 2001, through June 30, 2016). These data contain enrollee information (sex, race/ethnicity, age, and eligibility dates), diagnosis codes, and procedure codes. This study was determined to be exempt from review, and the requirement for obtaining patient written informed consent was waived by the Michigan Medicine Institutional Review Board because deidentified data were used. The study conforms to the International Society for Pharmacoeconomics and Outcomes Research (ISPOR) reporting guidelines for comparative-effectiveness studies.^27^

### Study Population

This study identified patients aged 5 to 24 years when diagnosed as having CD between July 1, 2001, and June 30, 2014. Excluded were children younger than 5 years at diagnosis (n = 93) because CD with very early onset often manifests differently and is more likely to represent an underlying immune defect with CD-like characteristics,^28,29,30,31^ and patients older than 24 years old at diagnosis (n = 8794) because pediatricians may provide care up to age 24 years. We considered the index date of CD diagnosis to be the first occurrence of any office-based evaluation and management, emergency consultation, or inpatient evaluation and management, consultation, or observation claim with a diagnosis of CD (eAppendix 1 in the Supplement). The dates of analysis were October 2017 to February 2020.

**Figure 1.**
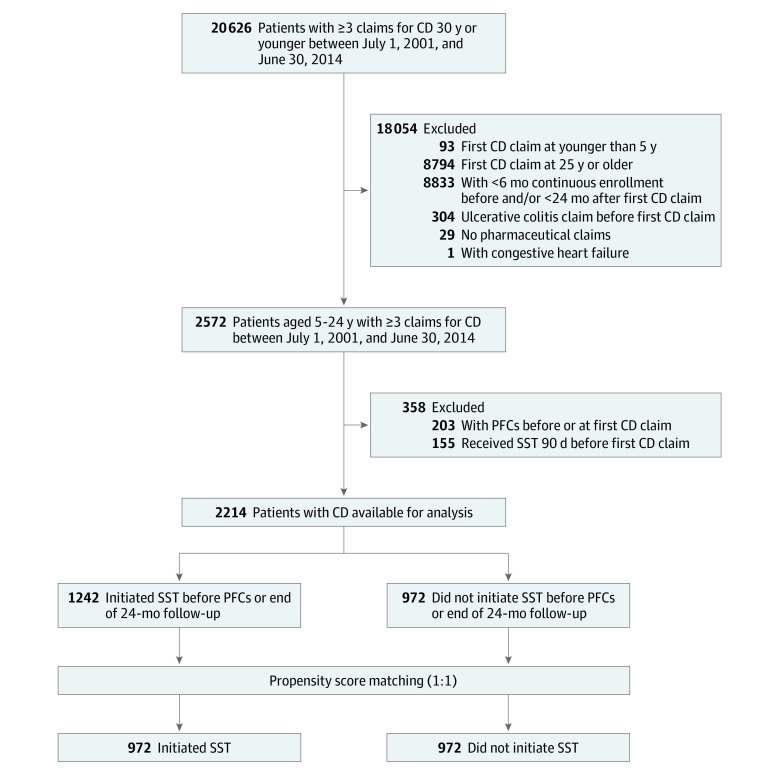
Selection of Study Patients CD indicates Crohn disease; PFCs, perianal fistulizing complications; and SST, steroid-sparing therapy.

Confirmatory CD diagnoses were required with at least 3 CD claims within 2 years of the index date.^32,33^ Patients were required to have at least 6 months of continuous insurance enrollment within any of the Optum included health plans before the index date (from January 1, 2001) and 2 years after the index date (through June 30, 2016) to capture treatments and outcomes. Patients who had ulcerative colitis diagnosis before CD diagnosis were excluded (n = 304) to avoid ambiguity of CD diagnosis. Because there was no pharmaceutical coverage indicator available in the data set, patients who did not have any prescription claims during their continuous enrollment period were considered as not having pharmaceutical coverage and were excluded from the study (n = 29). Because anti-TNFα medications are contraindicated in patients with congestive heart failure,^34^ patients with congestive heart failure before or at the time of CD diagnosis were excluded (n = 1). Patients were also excluded if they had a claim with perianal or genital fistula or abscess diagnosis, underwent perianal fistula–related surgical procedures before or at the index date (n = 203), or had immunomodulator or anti-TNFα use (n = 155) 90 days before the index date (Figure 1).

### Exposure

The exposure of interest was whether patients with CD initiated SST before development of PFCs. Steroid-sparing therapy was defined as immunomodulators (thiopurines and methotrexate) and/or anti-TNFα medications (infliximab, adalimumab, and certolizumab pegol) and was identified using the National Drug Code and the Healthcare Common Procedure Coding System codes reported in pharmacy or medical claims (eAppendix 1 in the Supplement). Because immunomodulators require about 90 days for full effectiveness, patients were considered as having initiated immunomodulators if they began the immunomodulators during the period between 90 days before the index date and 90 days before either developing PFCs or the end of study period. In contrast, anti-TNFα can take effect almost immediately, so patients were considered as having initiated anti-TNFα therapy if they began receiving anti-TNFα during the period between 90 days before the index date and before either developing PFCs or the end of the study period. SST was categorized as immunomodulators alone, anti-TNFα alone, or anti-TNFα plus immunomodulators.

### Outcomes

The primary outcome was PFC development within 2 years after the index date. Perianal fistulizing complication development was identified by previously validated claims-based case definition (perianal/genital fistula/abscess or seton/fistulotomy) using claims.^33^ The earliest date of PFC diagnosis or perianal complication–related surgical procedure was considered as the PFC date. Time from CD diagnosis to PFC development was calculated from the index date to the PFC date or to the end of the study period if no PFC development. There is no validated means of assessing complexity or severity of PFCs in administrative claims data. Consequently, we used ostomies (colostomy, ileostomy, or enterostomy) as markers of PFC severity because patients with complex or recalcitrant PFCs commonly require diverting ostomy to facilitate healing.^19^ Because ostomy can be performed for reasons other than PFCs, we limited the assessment to ostomy that first occurred in claims at or after PFC development but before the end of the 2-year study period.

### Covariates

Patient-level covariates included sex, race/ethnicity (categorized as white, black, Hispanic, or Asian individuals or unknown), age at CD diagnosis, education level of the primary insurance policyholder (categorized as high school or less, college or higher, or unknown), household income (categorized as <$40 000, $40 000-$49 999, $50 000-$59 999, $60 000-$74 999, $75 000-$99 999, ≥$100 000, or unknown), geographic region, and year of CD diagnosis. Comorbid conditions (eg, arthritis and gastrointestinal bleeding) were included to adjust for all potential confounders; these were identified through *International Classification of Diseases*, *Ninth Revision*, *Clinical Modification* diagnosis codes using medical or inpatient claims before or at the index date. Other medications were identified through the National Drug Code or Healthcare Common Procedure Coding System codes using pharmacy, medical, or inpatient claims data (including antibiotics and systemic corticosteroids) taken before the initiation of SST or PFC development or the end of the study period, whichever occurred first.

### Statistical Analysis

Descriptive analyses were conducted for all patients included in the study cohort. To assess the consequences of SST initiation in preventing PFC development within 2 years of the index date, propensity score matching was performed between patients who did or did not initiate SST to create a subcohort adjusted for all available potential confounders (eAppendix 2 in the Supplement). Covariate balance was checked before and after propensity score matching with χ^2^ tests. Crude 2-year rates of remaining free from PFCs after CD diagnosis were estimated for patients initiating and not initiating SST using the Kaplan-Meier method. Log-rank *P* < .05 was considered statistically significant. Cox proportional hazards multivariable regression analyses estimated hazard ratios (HRs) for PFC development from CD diagnosis and before the end of the study period. Graphical methods were used to assess proportional hazards assumption. Two-sided *P* < .05 was considered statistically significant. Several sensitivity analyses were conducted. First, because a selected medication type may have different effectiveness in preventing PFC development, a sensitivity analysis that included medication type was performed. Second, the association between SST and the risk of PFC development over a longer time horizon was also assessed using both a 3-year (among patients diagnosed as having CD between July 1, 2001, and June 30, 2013) and a 4-year (among patients diagnosed as having CD between July 1, 2001, and June 30, 2012) period after the index date. Most statistical analyses were performed using SAS, version 9.4 (SAS Institute Inc). Propensity score matching was performed, and Kaplan-Meier curves were generated with Stata, version 14.2 (StataCorp LLC).

## Results

### Study Cohort

The study identified 2214 patients aged 5 to 24 years who were diagnosed as having CD between 2001 and 2014 (Figure 1 and eTable 1 in the Supplement). The mean (SD) age at CD diagnosis was 17.0 (4.5) years, and 1151 (52.0%) were male. Most patients were white individuals (1739 [78.6%]). The primary insurance policyholder mostly had an education level of college or higher (1787 [80.7%]). The median enrollment time before the index date was 753 days (range, 183-4858 days), and the median follow-up was 1351 days (range, 730-5473 days).

### Medication Use

Among the cohort, 1242 patients (56.1%) initiated SST (778 [35.1%] immunomodulators alone, 192 [8.7%] anti-TNFα alone, and 272 [12.3%] anti-TNFα plus immunomodulators) before PFC development or by the end of the 2-year study period, and 972 patients (43.9%) did not (eTable 1 in the Supplement). There were differences in SST initiation by sex (560 of 1063 [52.7%] female individuals and 682 of 1151 [59.3%] male individuals used SST, *P* = .002) and by household income (higher income was associated with more SST use), but there were no differences in SST initiation by education level (1016 of 1242 [81.8%] with SST vs 771 of 972 [79.3%] without SST were college educated, *P* = .27). Patients who initiated SST tended to be younger at CD diagnosis (mean [SD] age, 16.3 [4.3] vs 17.8 [4.6] years; *P* < .001), were more likely to have anemia (214 of 1242 [17.2%] vs 106 of 972 [10.9%], *P* < .001), less commonly used antibiotics (513 of 1242 [41.3%] vs 594 of 972 [61.1%], *P* < .001), and more commonly used corticosteroids (798 of 1242 [64.3%] vs 492 of 972 [50.6%], *P* < .001) than non-SST users. Those who did not initiate SST were more likely than those who did to have history of cancer (33 of 972 [3.4%] vs 25 of 1242 [2.0%], *P* = .04) or liver disease (64 of 972 [6.6%] vs 57 of 1242 [4.6%], *P* = .04) before or at CD diagnosis. After propensity score matching, the SST group had a median continuous enrollment of 2495 days (range, 943-5659 days), with a median follow-up of 1380 days (range, 730-5473 days) after CD diagnosis. The non-SST group had a median continuous enrollment of 2358 days (range, 942-5659 days), with a median follow-up of 1321 days (range, 731-5099 days).

### Perianal Fistulizing Complications

**Figure 2.**
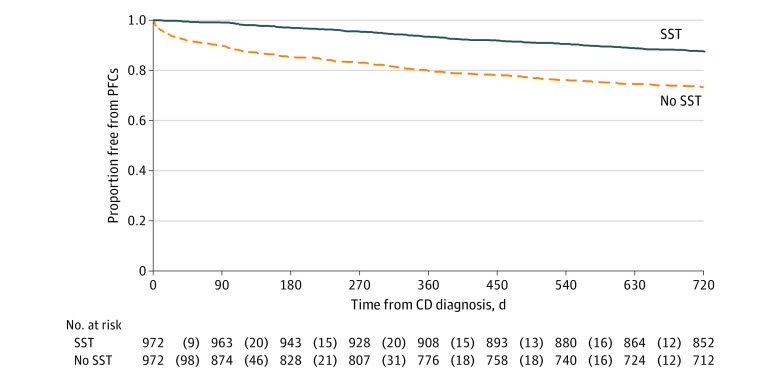
Proportion Free From Perianal Fistulizing Complications (PFCs) Among Propensity Score–Matched Subgroups After propensity score matching, patients who received steroid-sparing therapy (SST) were less likely than those who did not (no SST) to develop PFCs (hazard ratio, 0.41; 95% CI, 0.33-0.52; *P* < .001). Numbers in parentheses represent PFC events. Note that SST may have been started at any time after Crohn disease (CD) diagnosis but before PFC development or the end of the study. Graphical assessment indicates proportionality assumption was met.

Before propensity score matching, 415 of 2214 patients (18.7%) developed PFCs within 2 years of the index date. The crude 2-year rate of remaining free from PFCs was 73.1% (711 of 972) for patients who did not use SST vs 87.6% (1088 of 1242) for patients who initiated SST. After propensity score matching, 972 patients remained in each treatment group (Table 1). After propensity score matching, 384 of 1944 (19.8%) developed PFCs within 2 years of the index date. The crude 2-year rate of remaining free from PFCs was 73.1% (711 of 972) for patients who did not use SST vs 87.3% (849 of 972) for patients who initiated SST (log-rank *P* < .001) (Figure 2).

**Table 1.** Demographics of the Study Population After Propensity Score Matching

Variable	No. (%)		*P* value
	SST (n = 972)	No SST (n = 972)	
Sex			
Female	473 (48.7)	503 (51.8)	.17
Male	499 (51.3)	469 (48.3)	
Race/ethnicity			
White	761 (78.3)	750 (77.2)	.90
Black	69 (7.1)	74 (7.6)	
Hispanic	52 (5.4)	61 (6.3)	
Asian	20 (2.1)	20 (2.1)	
Unknown	70 (7.2)	67 (6.9)	
Age at CD diagnosis, mean (SD), y	17.2 (4.2)	17.8 (4.6)	<.001
No. of encounters in 2 y, mean (SD)	18.7 (1.7)	15.9 (10.5)	<.001
No. of CD encounters in 2 y, mean (SD)	11.5 (7.1)	7.5 (5.0)	<.001
Education level of the primary insurance policyholder			
High school or less	168 (17.3)	177 (18.2)	.57
College or higher	773 (79.5)	771 (79.3)	
Unknown	31 (3.2)	24 (2.5)	
Household income, $			
<40 000	63 (6.5)	65 (6.7)	.68
40 000-49 999	30 (3.1)	37 (3.8)	
50 000-59 999	33 (3.4)	36 (3.7)	
60 000-74 999	59 (6.1)	71 (7.3)	
75 000-99 999	107 (11.0)	110 (11.3)	
≥100 000	410 (42.2)	373 (38.4)	
Unknown	270 (27.8)	280 (28.8)	
Insurance product			
Exclusive provider organization	127 (13.1)	124 (12.8)	.61
Health maintenance organization	137 (14.1)	131 (13.5)	
Indemnity	0	1 (0.1)	
Other	2 (0.2)	1 (0.1)	
Point of service	652 (67.1)	645 (66.4)	
Preferred provider organization	54 (5.6)	70 (7.2)	
Comorbid conditions			
Anemia	131 (13.5)	106 (10.9)	.08
Arthritis			
Juvenile idiopathic	4 (0.4)	4 (0.4)	>.99
Spondyloarthropathy	5 (0.5)	5 (0.5)	>.99
Other	170 (17.5)	175 (18.0)	.77
Cancer	21 (2.2)	33 (3.4)	.10
Cardiovascular disease	27 (2.8)	33 (3.4)	.43
Pregnancy	18 (1.9)	27 (2.8)	.17
Genital inflammation	64 (6.6)	73 (7.5)	.43
Gastrointestinal bleeding	217 (22.3)	215 (22.1)	.91
Gastrointestinal obstruction	24 (2.5)	24 (2.5)	>.99
Infection			
Abscess	81 (8.3)	96 (9.9)	.24
Serious infection^a^	57 (5.9)	59 (6.1)	.85
Liver disease	51 (5.3)	64 (6.6)	.21
Internal fistula	6 (0.6)	4 (0.4)	.53
Other medications			
Antibiotics	504 (51.9)	594 (61.1)	<.001
Systemic corticosteroids	550 (56.6)	492 (50.6)	.008

Abbreviations: CD, Crohn disease; SST, steroid-sparing therapy.

^a^ Serious infections include meningitis, encephalitis, influenza, HIV, tuberculosis, and sepsis.

### Perianal Fistula Severity

In the propensity score–matched subgroups, 40 patients underwent ostomy after PFCs (10.4% of 384 patients who developed PFCs) during the 2-year study period. Of these, 7 had initiated SST (5.7% of 123 patients who initiated SST but still developed PFCs), and 33 did not use SST (12.6% of 261 patients who had not initiated SST and developed PFCs) (*P* = .04). Among those who developed PFCs, 55% fewer patients underwent ostomy after PFC if they had previously initiated SST compared with those who did not initiate SST.

### Patient Characteristics

After adjusting for sociodemographic and clinical characteristics (Table 2), the use of SST was associated with a 59% decreased risk of PFC development in the 2 years after CD diagnosis (HR, 0.41; 95% CI, 0.33-0.52; *P* < .001) compared with no SST. Antibiotic use was associated with a 23% lower risk of developing PFCs (HR, 0.77; 95% CI, 0.62-0.96; *P* = .02). For each additional year of age at CD diagnosis, the risk of developing PFCs increased 5% (HR, 1.05; 95% CI, 1.02-1.07; *P* < .001). Compared with patients whose primary insurance policyholder had a high school or less education level, patients whose primary insurance policyholder had a college or higher education level had a 27% lower risk of PFC development (HR, 0.73; 95% CI, 0.56-0.95; *P* = .02). Patients with internal fistulas had an almost 3-fold greater increased risk of developing PFCs (HR, 2.98; 95% CI, 1.07-8.33; *P* = .04). Patients with gastrointestinal bleeding had a 33% greater risk of developing PFCs (HR, 1.33; 95% CI, 1.05-1.68; *P* = .02). Other patient characteristics were not associated with risk of PFC development (Table 2).

**Table 2.** Multivariable Cox Proportional Hazards Model for Perianal Fistulizing Complications Among Users of Steroid-Sparing Therapy (SST) Compared With Matched Nonusers^a^

Variable	HR (95% CI)	*P* value
SST		
SST	0.41 (0.33-0.52)	<.001
No SST	1 [Reference]	NA
Sex		
Female	1.02 (0.83-1.26)	.85
Male	1 [Reference]	NA
Race/ethnicity		
White	1 [Reference]	NA
Black	0.99 (0.67-1.46)	.95
Hispanic	0.85 (0.54-1.33)	.48
Asian	0.81 (0.36-1.83)	.61
Unknown	1.02 (0.64-1.63)	.93
Age at CD diagnosis, per year	1.05 (1.02-1.07)	<.001
Education level of the primary insurance policyholder		
High school or less	1 [Reference]	NA
College or higher	0.73 (0.56-0.95)	.02
Unknown	0.70 (0.31-1.59)	.40
Household income,$		
<40 000	1 [Reference]	NA
40 000-49 999	0.94 (0.52-1.71)	.83
50 000-59 999	0.89 (0.47-1.69)	.72
60 000-74 999	0.80 (0.48-1.36)	.41
75 000-99 999	0.88 (0.55-1.41)	.60
≥100 000	0.91 (0.60-1.39)	.66
Unknown	0.86 (0.56-1.32)	.49
Comorbid conditions		
Anemia	1.11 (0.82-1.52)	.49
Arthritis, all types	1.07 (0.82-1.39)	.61
Cancer	0.91 (0.51-1.65)	.76
Cardiovascular disease	0.88 (0.47-1.64)	.69
Pregnancy	1.36 (0.78-2.37)	.28
Genital inflammation	1.01 (0.69-1.47)	.97
Gastrointestinal bleeding	1.33 (1.05-1.68)	.02
Gastrointestinal obstruction	0.54 (0.24-1.24)	.15
Infection		
Abscess	1.16 (0.83-1.63)	.39
Serious infection^b^	0.86 (0.54-1.37)	.52
Liver disease	1.10 (0.73-1.66)	.64
Internal fistula	2.98 (1.07-8.33)	.04
Other medications		
Antibiotics	0.77 (0.62-0.96)	.02
Systemic corticosteroids	0.85 (0.69-1.06)	.14

Abbreviations: CD, Crohn disease; HR, hazard ratio; NA, not applicable.

^a^ The model also adjusted for year at diagnosis and geographic region.

^b^ Serious infections include meningitis, encephalitis, influenza, HIV, tuberculosis, and sepsis.

### Sensitivity Analyses

To further understand whether the risk of developing PFCs varied by SST medication type, additional analyses of medication type were conducted (eTable 2 in the Supplement). Compared with no SST, the use of immunomodulators alone was associated with a 52% reduction in the risk of 2-year PFC development (HR, 0.48; 95% CI, 0.37-0.62; *P* < .001), anti-TNFα alone was associated with a 47% lower risk of developing PFCs (HR, 0.53; 95% CI, 0.36-0.78; *P* = .001), and using both immunomodulators and anti-TNFα (combination therapy) was associated with an 83% lower risk of developing PFCs (HR, 0.17; 95% CI, 0.09-0.30; *P* < .001).

When evaluating longer follow-up periods, results of both 3-year and 4-year time frames were similar to our original findings (eTable 3 in the Supplement). Steroid-sparing therapy was associated with a 56% decreased risk of PFC development within 3 years after CD diagnosis (HR, 0.44; 95% CI, 0.36-0.54; *P* < .001) and a 52% decreased risk of PFC development within 4 years (HR, 0.48; 95% CI, 0.39-0.59; *P* < .001). Because patient insurance coverage after age 18 years may have changed, we performed additional sensitivity analysis limiting the population to patients 18 years or younger at CD diagnosis. After propensity score matching, 484 individuals were left in each treatment group. In this younger cohort, we similarly found that SST use was associated with a 50% reduction in risk of PFCs within 2 years (HR, 0.50; 95% CI, 0.37-0.69; *P* < .001).

After propensity score matching and adjusting for other covariates, an era association was found, with a 43% increased risk of PFC development in patients diagnosed as having CD in 2009 to 2014 compared with 2001 to 2005 (HR, 1.43; 95% CI, 1.08-1.88; *P* = .01). To address the possibility of practice change after the US Food and Drug Administration approved infliximab and adalimumab use for pediatric CD, we performed an additional sensitivity analysis limiting the study time frame to the years starting in 2006, when the US Food and Drug Administration approved infliximab.^35^ We found a suggestion of a similar but not statistically significant pattern of a 29% increased risk of PFC development in 2009 to 2014 compared with 2006 to 2008 (HR, 1.29; 95% CI, 0.99-1.68; *P* = .06). Looking back at other changes across these periods, there were more patients excluded because PFCs were found at or before CD diagnosis in the later years of the study. Over time, the proportion of patients with PFCs present at or before CD diagnosis increased from 6.3% (49 of 779) in 2001 to 2005 to 6.6% (39 of 590) in 2006 to 2008 and to 9.6% (115 of 1203) in 2009 to 2014.

## Discussion

This study used a large, privately insured cohort to investigate the effectiveness of SST for reducing the risk of PFC development among young patients with CD. Consistent with prior pediatric evidence,^7^ we found that almost 1 in 5 patients who were initially free from PFCs at CD diagnosis developed PFCs within the following 2 years. We noted that the use of SST was less common than guidelines recommend^36^; however, using a propensity score–matched cohort, we found that introduction of SST was associated with a 59% lower risk of developing PFCs among young patients with newly diagnosed CD. We also found evidence that SST use may reduce the severity or complexity of PFCs if they do develop. Among patients who developed PFCs, a smaller proportion of those who had previously initiated SST subsequently underwent diverting ostomy compared with those who had not initiated SST before PFC development.

These are important findings because PFCs can be devastating, with long-lasting major negative consequences on QOL.^14,15^ They can cause pain, feculent drainage, fecal incontinence, and dyspareunia and are often associated with negative body image and depression.^15,37,38^ Perianal fistulizing complications are difficult to treat; they often require invasive surgical procedures but still commonly reoccur.^5,39,40^ Despite optimal medical and/or surgical therapy, there remains a substantial risk of requiring a permanent ostomy. Therefore, evidence-based strategies for preventing PFC development among patients with CD are greatly needed.

Although ample evidence exists about the effectiveness of various medical therapies for treatment of PFCs because of CD,^16,40^ this investigation is the first study, to our knowledge, that directly evaluates whether PFCs may be preventable. A small study^41^ provided preliminary indications that perianal fistulas may be preventable. In that open randomized trial, combined immunosuppression was associated with improved outcomes compared with routine care (n = 133), and fewer patients developed perianal fistulas in the treatment group (not statistically significant). However, in post hoc analysis of patients who underwent follow-up endoscopy (n = 46), fewer patients who achieved mucosal healing developed perianal fistulas, suggesting that PFCs may be preventable if CD is well treated.^42^ Although mucosal healing is not captured in administrative claims data, our study similarly found that patients treated with SST (more effective than non-SST^21,22,43^) and especially with combination therapy (more effective than either immunomodulators or anti-TNFα alone^22,41,43^) were less likely to develop PFCs.

It was particularly notable herein that PFCs appear to be preventable even among subgroups of patients at higher risk of developing them. Perianal fistulizing complications have previously been found to develop more commonly among black and Asian patients than among white patients.^7,25,44,45^ After adjusting for patient characteristics and medical therapies, we found no differences in PFC development by race/ethnicity. This finding suggests that previously identified racial/ethnic disparities^7,25,44,46^ may not be attributable to biological differences between racial/ethnic groups but rather may be associated with disparities in access or other aspects of care.

It is unclear why the rate of PFC development was higher in the latter years of the study. We noted that an increasing proportion of patients with PFCs present before or at CD diagnosis over the years. There is no clear reason why the behavior of CD should be changing over time. We hypothesize that this finding indicates greater delay in CD diagnosis, which may result in patients being sicker and having more aggressive disease at the time of CD diagnosis, thus increasing the likelihood of developing disease complications, such as PFCs. However, understanding these changes over time will require further investigation.

### Limitations

This study has important limitations to acknowledge. As with all retrospective studies, there is potential for confounding by indication. This risk can be mitigated by propensity score methods, but they cannot completely remove all potential confounders. Other important limitations relate to a lack of clinical information in administrative claims data. As a consequence, we were unable to assess CD location or endoscopic severity or whether patients or their family members were smokers. Propensity score–matched analyses may minimize the risk of more smokers in one group than the other but cannot fully obviate this problem. Another potential limitation of this study is that we excluded patients without any recorded prescriptions because they may have had no pharmacy benefits. It is also possible that some patients who did not take any prescription medications but instead used diet or herbal therapies may have been mistakenly excluded for appearing to have no pharmacy benefits. We could not detect the consequences of these treatments with our study design. These may be important contributors or confounders that will require further examination in prospective studies. Despite these limitations, this study was rigorously conducted and presents the first compelling evidence to date that PFCs may be preventable with the use of SST.

## Conclusions

Among young patients with CD, those who initiated SST were 59% less likely to develop PFCs than those who were not treated with SST in the 2 years after CD diagnosis. Furthermore, among those who developed PFCs, fewer SST users underwent ostomy than SST nonusers. These results indicate that PFCs may be preventable or less severe with effective medical therapy. We also found that the use of SST was lower than expected, being used by only slightly more than half of young patients with CD who did not have PFCs at diagnosis. The reason for this infrequent use of medications that are considered standard of care is unknown and requires further investigation. The study findings support existing guidelines, which recommend SST use for all patients with CD.^36,47,48,49^ Most important, we believe that these results provide an evidence base on which to develop strategies for prevention of PFCs in young patients with CD.

## Notes

Supplement. **eAppendix 1.** Administrative Claims Definitions **eAppendix 2.** Propensity Score Matching Model **eTable 1.** Demographic Information for Patients Before Propensity Score Matching **eTable 2.** Multivariate Cox Proportional Hazards Model for Perianal Fistulizing Complications Among Users of Steroid-Sparing Therapies Compared With Matched Nonusers (N = 1944) **eTable 3.** Multivariate Cox Proportional Hazards Model for Perianal Fistulizing Complications Among Users of Steroid-Sparing Therapies Compared With Matched Nonusers in 3-Year and 4-Year Follow-up
