# Supplementary material for: Association Between Steroid-Sparing Therapy and the Risk of Perianal Fistulizing Complications Among Young Patients With Crohn Disease
Source: JAMA Netw Open. 2020 Jun 9;3(6):e207378. doi: 10.1001/jamanetworkopen.2020.7378 (PMC7284306; doi:10.1001/jamanetworkopen.2020.7378)
Supplement: Supplement. — eAppendix 1. Administrative Claims Definitions eAppendix 2. Propensity Score Matching Model eTable 1. Demographic Information for Patients Before Propensity Score Matching eTable 2. Multivariate Cox Proportional Hazards Model for Perianal Fistulizing Complications Among Users of Steroid-Sparing Therapies Compared With Matched Nonusers (N = 1944) eTable 3. Multivariate Cox Proportional Hazards Model for Perianal Fistulizing Complications Among Users of Steroid-Sparing Therapies Compared With Matched Nonusers in 3-Year and 4-Year Follow-up [file jamanetwopen-3-e207378-s001.pdf]

Supplementary Online Content

Adler J, Lin CC, Gadepalli SK, Dombkowski KJ. Association between steroid-sparing therapy and the risk of perianal fistulizing complications among young patients with Crohn disease. *JAMA Netw Open*. 2020;3(6):e207378. doi:10.1001/jamanetworkopen.2020.7378

eAppendix 1. Administrative Claims Definitions

eAppendix 2. Propensity Score Matching Model

eTable 1. Demographic Information for Patients Before Propensity Score Matching

eTable 2. Multivariate Cox Proportional Hazards Model for Perianal Fistulizing Complications Among Users of Steroid-Sparing Therapies Compared With Matched Nonusers (N=1944)

eTable 3. Multivariate Cox Proportional Hazards Model for Perianal Fistulizing Complications Among Users of Steroid-Sparing Therapies Compared With Matched Nonusers in 3-Year and 4-Year Follow-up

This supplementary material has been provided by the authors to give readers additional information about their work.

© 2020 Adler J et al. *JAMA Network Open*.

eAppendix 1. Administrative claims definitions

CPT: Current Procedural Terminology Code
ICD: International Classification of Disease Diagnosis Code

Evaluation and management services (including consultation and observation)

1. Office-based: CPT: 99201-99205, 99241-99245, 99211-99215
2. Inpatient: CPT: 99221-99226, 99234-99236, 99218-99220, 99231-99233, 99238-99239, 99251-99255
3. Emergency Room: CPT: 99281-99285

Crohn's disease diagnoses: ICD-9-CM 555.x or ICD-10-CM K50.x
Ulcerative colitis diagnoses: ICD-9-CM 556.x or ICD-10-CM K51.x, K52.3

Perianal fistula diagnoses

1. Perianal fistula:
    a. ICD-9-CM: 565.1
    b. ICD-10-CM: K60.3, K60.4, K60.5
2. Genital fistula:
    a. ICD-9-CM: 619.1, 619.2, 619.8, 619.9, 608.89, 607.89, 599.1
    b. ICD-10-CM: N49.9; N82.x, excluding N82.0 and N82.1
3. Perirectal abscess:
    a. ICD-9-CM: 566.x, 682.5
    b. ICD-10-CM: K61.0 – K61.4
4. Genital abscess:
    a. ICD-9-CM: 616.1, 616.4, 614.4, 567.0, 601.2, 593.89, 597.0, 595.89, 608.4
    b. ICD-10-CM: N75.1, 76.4, N49.8, N49.9, N48.2, N49.2
5. Perianal lesions:
    a. ICD-9-CM: 455.9, 569.49, 455.x
    b. ICD-10-CM: K60.0, K60.1, K60.2, K62.81, K64.4, K62.6, K62.9, K64.X
6. Genital lesions:
    a. ICD-9-CM: 616.50, 616.51, 608.89
    b. ICD-10-CM: N77.0, 76.5, 76.6, N48.5, N34.2, N47.4, N50.89

Perianal fistula-related surgical procedures

a. Seton placement:
    a. CPT: 46020
b. Seton removal:
    a. CPT: 46030,
    b. ICD-9-CM: 49.93
c. Fistulotomy or Fistulectomy:
    a. CPT: 46270, 46275, 46280, 46285
    b. ICD-9-CM: 49.11, 49.12
d. I&D perianal abscess:
    a. CPT: 46040, 46045-51, 46060, 46000, 46005, 45020
    b. ICD-9-CM: 49.1, 49.01
e. Perianal lesion removal:
    a. CPT: 46220, 46230, 46900, 46910, 46916, 46917, 46922, 46924
    b. ICD-9-CM: 49.02, 49.03, 49.04, 49.39
f. Hemorrhoid procedure:
    a. CPT: 46221, 46230, 46250, 46255, 46257, 46258, 46260, 46261, 46262, 46320, 46934, 46935, 46936, 46500, 45398
    b. ICD-9-CM: 49.45, 49.46
g. Perianal repair procedures:
    a. CPT: 46288, 46706, 46707, 46280

© 2020 Adler J et al. *JAMA Network Open*.

Ostomy

a. ICD-9-CM: 46.x
b. CPT: 44141, 44143, 44144, 44145, 44146, 44150, 44151, 44155, 44156, 44157, 44158, 44160, 44187, 44188, 44205, 44206, 44207, 44208, 44210, 44211, 44212, 44310

All medication utilization was identified through HCPCS codes or NDC codes. The list of NDC codes would be available upon request.

Immuomodulator

a. Methotrexate
    a. HCPCS: J9260 (50mg), J9250 (5mg), J8610 (oral 2.5 mg)
b. Mercaptopurine
    a. HCPCS: S0108 (oral 50mg)
c. Azathioprine
    a. HCPCS: J7500 (oral 50mg), J7501 (100mg)

Anti-TNF

a. Infliximab
    a. HCPCS: J1745 (exclude biosimilar), Q5102 (biosimilar), Q5103 (biosimilar), Q5104 (biosimilar), Q5109 (biosimilar)
b. Adalimumab
    a. HCPCS: J0135 (20mg)
c. Certolizumab
    a. HCPCS: J0717 (1mg)
d. Golimumab
    a. HCPCS: J1602 (1mg)

Antibiotics

a. Metronidazole
    a. HCPCS: S0030 (500mg)
b. Ciprofloxacin
    a. HCPCS: J0744 (200mg)
c. Levofloxacin
    a. HCPCS: J1956 (250mg)

Steroid

a. Methylprednisolone
    a. HCPCS: J1020, J1030, J1040, J2920, J2930, J2650
b. Dexamethasone
    a. HCPCS: J1094, J1100, J7637, J7638, S0173, J7312
c. Hydrocortisone
    a. HCPCS: J1700, J1710, J1720
d. ICD-9-procedure code: 99.23

© 2020 Adler J et al. *JAMA Network Open*.

eAppendix 2. Propensity-score matching model

Logistic regression model in predicting SST initiation before PFC
SST=age male race education income_level geographic_division diagnosis_year anemia arthritis_other arthritis_spondylo bleeding cancer cardiovascular jia infection_abscess infection_serious liver_disease internal_fistula antibiotics steroid

Predict propensity score and then match between No SST patients and SST patients

© 2020 Adler J et al. *JAMA Network Open*.

eTable 1. Demographic Information of Patients Before Propensity Score Matching

Variable	Total (n = 2214)	No Steroid-Sparing Therapy (n = 972)	Steroid Sparing-Therapy (n = 1242)	p-value
	No. (Column %)	No. (Column %)	No. (Column %)	
Sex				
Female	1063 (48.0)	503 (51.7)	560 (45.1)	0.002
Male	1151 (52.0)	469 (48.3)	682 (54.9)	
Race / Ethnicity				
White	1739 (78.6)	750 (77.2)	989 (79.6)	0.26
Black	152 (6.9)	74 (7.6)	78 (6.3)	
Hispanic	117 (5.3)	61 (6.3)	56 (4.5)	
Asian	47 (2.1)	20 (2.1)	27 (2.2)	
Unknown	159 (7.2)	67 (6.9)	92 (7.4)	
Age at diagnosis, mean (sd), y	17.0 (4.5)	17.8 (4.6)	16.3 (4.3)	< 0.001
Number of encounters in 2 yr, mean(sd)	17.5 (10.7)	15.9 (10.5)	18.7 (10.7)	<0.001
Number of CD encounters in 2 yr, mean(sd)	9.7 (6.4)	7.1 (5)	11.3 (6.9)	<0.001
Education of primary insurance policy holder				
High school or less	371 (16.8)	177 (18.2)	194 (15.6)	0.27
College or higher	1787 (80.7)	771 (79.3)	1016 (81.8)	
Unknown	56 (2.5)	24 (2.5)	32 (2.6)	
Household Income				
<$40k	138 (6.2)	65 (6.7)	73 (5.9)	0.006
$40K-$49K	67 (3.0)	37 (3.8)	30 (2.4)	
$50K-$59K	81 (3.7)	36 (3.7)	45 (3.6)	
$60K-$74K	139 (6.3)	71 (7.3)	68 (5.5)	
$75K-$99K	233 (10.5)	110 (11.3)	123 (9.9)	
$100K+	952 (43.0)	373 (38.4)	579 (46.6)	
Unknown	604 (27.3)	280 (28.8)	324 (26.1)	
Comorbid conditions				
Anemia	320 (14.5)	106 (10.9)	214 (17.2)	<.001
Arthritis				
Juvenile idiopathic	17 (0.8)	4 (0.4)	13 (1.1)	0.09
Spondyloarthropathy	11 (0.5)	5 (0.5)	6 (0.5)	0.92
Other	401 (18.1)	175 (18.0)	226 (18.2)	0.91
Cancer	58 (2.6)	33 (3.4)	25 (2.0)	0.04
Cardiovascular disease	69 (3.1)	33 (3.4)	36 (2.9)	0.50
Pregnancy	47 (2.1)	27 (2.8)	20 (1.6)	0.06
Gastrointestinal bleeding	489 (22.1)	215 (22.1)	274 (22.1)	0.97
Gastrointestinal obstruction	54 (2.4)	24 (2.5)	30 (2.4)	0.94
Genital inflammation	147 (6.6)	73 (7.5)	74 (6.0)	0.15
Infection				
Abscess	193 (8.7)	96 (9.9)	97 (7.8)	0.09
Serious infection	129 (5.8)	59 (6.1)	70 (5.6)	0.67
Liver disease	121 (5.5)	64 (6.6)	57 (4.6)	0.04
Internal fistula	10 (0.5)	4 (0.4)	6 (0.5)	0.80
Other medications				
Antibiotics	1107 (50.0)	594 (61.1)	513 (41.3)	<0.001
Steroid	1290 (58.3)	492 (50.6)	798 (64.3)	<0.001

No, number of patients; sd, standard deviation.

© 2020 Adler J et al. *JAMA Network Open*.

eTable 2. Multivariable Cox Proportional Hazard Model for Perianal Fistulizing Complication among Users of Steroid-Sparing Therapies Compared with Matched Non-Users (N=1,944)

Variable	Hazard Ratio (95%CI)	p-value
Steroid-sparing therapy		
No SST	1.00*	
Immunomodulator	0.48 (0.37-0.62)	< 0.001
Anti-TNFα	0.53 (0.36-0.78)	0.001
Anti-TNFα + Immunomodulator	0.17 (0.09-0.30)	< 0.001
Sex		
Female	1.03 (0.83-1.27)	0.81
Male	1.00*	
Race		
White	1.00*	
Black	0.95 (0.64-1.41)	0.81
Hispanic	0.85 (0.54-1.33)	0.47
Asian	0.78 (0.35-1.77)	0.56
Unknown	1.04 (0.65-1.65)	0.88
Age at diagnosis, per year	1.05 (1.02-1.08)	<0.001
Education of primary insurance policy holder		
High school or less	1.00*	
College or higher	0.72 (0.56-0.95)	0.02
Unknown	0.70 (0.31-1.59)	0.40
Household Income		
<$40k	1.00*	
$40K-$49K	0.96 (0.53-1.74)	0.88
$50K-$59K	0.88 (0.46-1.67)	0.70
$60K-$74K	0.83 (0.49-1.39)	0.47
$75K-$99K	0.90 (0.57-1.44)	0.66
$100K+	0.91 (0.60-1.39)	0.67
Unknown	0.86 (0.56-1.32)	0.49
Comorbid conditions		
Anemia	1.14 (0.84-1.55)	0.42
Arthritis (all types)	1.07 (0.82-1.39)	0.64
Cancer	0.90 (0.50-1.63)	0.73
Cardiovascular disease	0.86 (0.46-1.59)	0.62
Pregnancy	1.29 (0.74-2.26)	0.37
Genital inflammation	1.02 (0.70-1.49)	0.92
Gastrointestinal bleeding	1.34 (1.06-1.69)	0.01
Gastrointestinal obstruction	0.55 (0.24-1.27)	0.16
Infection		
Abscess	1.18 (0.84-1.65)	0.35
Serious infection†	0.89 (0.56-1.42)	0.64
Liver disease	1.10 (0.73-1.67)	0.64
Internal fistula	3.20 (1.15-8.94)	0.03
Other Medications		
Antibiotics	0.77 (0.62-0.96)	0.02
Steroid	0.85 (0.69-1.05)	0.13

SST, steroid-sparing therapy; *Reference group; †Serious infections include meningitis, encephalitis, influenza, HIV, tuberculosis, sepsis. Model also adjusted for year at diagnosis, geographic region.

© 2020 Adler J et al. *JAMA Network Open*.

eTable 3. Multivariable Cox Proportional Hazard Model for Perianal Fistulizing Complication among Users of Steroid-Sparing Therapies Compared with Matched Non-Users in 3-year and 4-year follow up

	Subgroups with 3-year follow-up (n = 1,722)		Subgroups with 4-year follow-up (n = 1,516)	
**Variable**	**Hazard ratio (95%CI)**	**p-value**	**Hazard ratio (95%CI)**	**p-value**
Steroid-sparing therapy				
No SST	1.00*		1.00*	
SST	0.44 (0.36-0.54)	<0.001	0.48 (0.39-0.59)	<0.001
Sex				
Female	1.03 (0.84-1.27)	0.76	1.12 (0.91-1.38)	0.29
Male	1.00*		1.00*	
Race				
White	1.00*		1.00*	
Black	1.11 (0.77-1.59)	0.57	1.15 (0.80-1.65)	0.45
Hispanic	0.89 (0.59-1.36)	0.60	1.11 (0.74-1.68)	0.62
Asian	0.55 (0.22-1.34)	0.19	0.74 (0.33-1.68)	0.48
Unknown	1.03 (0.65-1.62)	0.92	1.20 (0.76-1.91)	0.43
Age at diagnosis, per year	1.05 (1.02-1.07)	<0.001	1.04 (1.01-1.06)	0.002
Education of primary insurance policy holder				
High school or less	1.00*		1.00*	
College or higher	0.73 (0.57-0.95)	0.02	0.73 (0.56-0.96)	0.02
Unknown	0.73 (0.34-1.58)	0.43	0.54 (0.25-1.18)	0.12
Household Income				
<$40k	1.00*		1.00*	
$40K-$49K	1.18 (0.67-2.08)	0.57	1.24 (0.71-2.17)	0.45
$50K-$59K	0.97 (0.52-1.82)	0.93	1.05 (0.55-1.98)	0.89
$60K-$74K	0.78 (0.45-1.34)	0.36	0.87 (0.50-1.51)	0.61
$75K-$99K	1.00 (0.64-1.59)	0.99	1.19 (0.74-1.91)	0.47
$100K+	1.07 (0.70-1.63)	0.75	1.28 (0.83-1.98)	0.26
Unknown	0.98 (0.64-1.50)	0.92	0.94 (0.61-1.45)	0.77
Comorbid conditions				
Anemia	1.13 (0.84-1.52)	0.42	1.06 (0.78-1.45)	0.70
Arthritis (all types)	1.00 (0.77-1.31)	0.97	0.92 (0.70-1.21)	0.55
Cancer	1.02 (0.59-1.75)	0.94	1.00 (0.58-1.72)	0.99
Cardiovascular disease	1.07 (0.61-1.86)	0.82	0.88 (0.49-1.56)	0.66
Pregnancy	1.60 (0.95-2.70)	0.08	1.63 (0.95-2.79)	0.08
Genital inflammation	1.10 (0.77-1.56)	0.62	1.09 (0.76-1.55)	0.64
Gastrointestinal bleeding	1.24 (0.99-1.56)	0.06	1.19 (0.95-1.51)	0.14
Gastrointestinal obstruction	0.89 (0.47-1.68)	0.72	0.86 (0.46-1.64)	0.65
Infection				
Abscess	1.29 (0.94-1.76)	0.11	1.26 (0.92-1.73)	0.16
Serious infection†	0.90 (0.58-1.39)	0.64	0.61 (0.37-1.02)	0.06
Liver disease	1.02 (0.68-1.53)	0.94	1.02 (0.65-1.60)	0.92
Internal fistula	2.65 (1.03-6.82)	0.04	3.55 (1.49-8.46)	0.004
Other Medications				
Antibiotics	0.72 (0.58-0.89)	0.002	0.68 (0.55-0.85)	<.001
Steroids	0.81 (0.66-0.99)	0.04	0.79 (0.64-0.97)	0.02

SST, steroid-sparing therapy; *Reference group; †Serious infections include meningitis, encephalitis, influenza, HIV, tuberculosis, sepsis. Model also adjusted for year of diagnosis and geographic region.

© 2020 Adler J et al. *JAMA Network Open*.
